# Peripheral markers of thyroid function: the effect of T_4_ monotherapy vs T_4_/T_3_ combination therapy in hypothyroid subjects in a randomized crossover study

**DOI:** 10.1530/EC-12-0064

**Published:** 2013-02-08

**Authors:** Ulla Schmidt, Birte Nygaard, Ebbe Winther Jensen, Jan Kvetny, Anne Jarløv, Jens Faber

**Affiliations:** 1 Endocrine Unit, Department of Medicine O Herlev University Hospital Herlev RingvejDK-2730, Herlev Denmark; 2 Department of Medicine Naestved Hospital Naestved Denmark; 3 Endocrine Unit, Department of Medicine Frederiksberg Hospital Herlev Denmark; 4 Faculty of Health Sciences University of Copenhagen Copenhagen Denmark

**Keywords:** Thyroid hormones, substitution, extrathyroidal effects

## Abstract

**Background:**

A recent randomized controlled trial suggests that hypothyroid subjects may find levothyroxine (l-T_4_) and levotriiodothyronine combination therapy to be superior to l-T_4_ monotherapy in terms of quality of life, suggesting that the brain registered increased T_3_ availability during the combination therapy.

**Hypothesis:**

Peripheral tissue might also be stimulated during T_4_/T_3_ combination therapy compared with T_4_ monotherapy.

**Methods:**

Serum levels of sex hormone-binding globulin (SHBG), pro-collagen-1-N-terminal peptide (PINP), and N-terminal pro-brain natriuretic peptide (NT-proBNP) (representing hepatocyte, osteoblast, and cardiomyocyte stimulation respectively) were measured in 26 hypothyroid subjects in a double-blind, randomized, crossover trial, which compared the replacement therapy with T_4_/T_3_ in combination (50 μg T_4_ was substituted with 20 μg T_3_) to T_4_ alone (once daily regimens). This was performed to obtain unaltered serum TSH levels during the trial and between the two treatment groups. Blood sampling was performed 24 h after the last intake of thyroid hormone medication.

**Results:**

TSH remained unaltered between the groups ((median) 0.83 vs 1.18 mU/l in T_4_/T_3_ combination and T_4_ monotherapy respectively; *P*=0.534). SHBG increased from (median) 75 nmol/l at baseline to 83 nmol/l in the T_4_/T_3_ group (*P*=0.015) but remained unaltered in the T_4_ group (67 nmol/l); thus, it was higher in the T_4_/T_3_ vs T_4_ group (*P*=0.041). PINP levels were higher in the T_4_/T_3_ therapy (48 vs 40 μg/l (*P*<0.001)). NT-proBNP did not differ between the groups.

**Conclusions:**

T_4_/T_3_ combination therapy in hypothyroidism seems to have more metabolic effects than the T_4_ monotherapy.

## Introduction

Triiodothyronine (T_3_) is regarded as the main metabolic active thyroid hormone. Available intracellular T_3_ is dependent on the transport of T_3_ from circulation as well as intracellular deiodination of thyroxine (T_4_) (1). In healthy euthyroid subjects, ∼20% of T_3_ is derived from thyroidal secretion and the remaining from local production (2). By contrast, hypothyroid subjects substituted with levothyroxine (l-T_4_) monotherapy demonstrated higher plasma T_4_/T_3_ ratio due to lack of thyroidal secretion of T_3_ (3). This might implicate different thyroid hormone actions at the cellular level in healthy euthyroid subjects compared with T_4_-substituted hypothyroid patients. In animal studies, T_4_ replacement therapy does not seem to result in adequate T_3_ concentrations in all tissues when compared with those obtained during T_4_/T_3_ combination therapy (4, 5).

Many studies have compared the effect of conventional T_4_ monotherapy to T_4_/T_3_ combination therapy in substituted hypothyroid subjects and have concluded that T_4_/T_3_ combination therapy is not beneficial (6). However, several of these studies have compared them without obtaining similar levels of serum TSH (7, 8). In a recent double-blind, randomized crossover trial, we compared the effect of two regimens in patients with hypothyroidism with the intention of keeping TSH stable and comparable within the two treatment groups (7). We found that T_4_/T_3_ combination therapy is superior to T_4_ monotherapy with respect to quality of life (QOL), depression and anxiety rating scales, and the patients' own preference. Our results indicate that peripheral extra-pituitary tissues can register the addition of T_3_ to the substitution regimen. The European Thyroid Association has recently published guidelines concerning T_3_ treatment of hypothyroidism and has concluded that T_4_/T_3_ combination therapy should be considered in some situations, but as an experimental modality (8). A recent clinical review on the same matter encourages further studies (9).

We consequently questioned whether peripheral tissues react differently to T_4_/T_3_ combination therapy than to conventional T_4_ monotherapy. Thus, markers of peripheral tissue function, which are known to be sensitive to changes in thyroid function, were studied (10, 11, 12, 13). The markers include sex hormone-binding globulin (SHBG) representing liver function, N-terminal pro-brain natriuretic peptide (NT-proBNP) representing cardiac function, and pro-collagen-1-N-terminal peptide (PINP) representing collagen production during bone formation.

## Materials and methods

### Subjects

This study has been described in detail previously (7). Briefly, the design was a double-blind, randomized, crossover study using block-randomization. In the first 12 weeks, 50 μg of the usual T_4_ dose was replaced with either 20 μg T_3_ or 50 μg T_4_ (tablets were identical), followed by a crossover for another 12 weeks. Due to the short half-life period of T_3_ and the risk of precipitating overt hypothyroidism, no washout period was included. The T_4_ dose was regulated if needed to withhold steady serum TSH levels. All patients were treated in each arm for exactly 12 weeks.

Inclusion criteria of the patients are i) overt, spontaneous hypothyroidism with serum TSH levels >20 mU/l, serum T_4_<60 nmol/l, and positive thyroid peroxidase antibodies (>60 U/ml) at the time of diagnosis; ii) euthyroidism at the time of screening, including unaltered T_4_ substitution for at least 6 months; and iii) age between 18 and 76 years. Exclusion criteria are i) pregnancy or planning pregnancy, ii) patients with any other chronic disease, iii) any previous T_3_ treatment, iv) active *post partum* thyroiditis, and v) hypothyroidism due to surgery or radioiodine treatment.

Patients included in this study were recruited from the same center, and blood samples were collected in the fasting state and before the intake of medicine (i.e. 24 h after the last intake of medication) at baseline, at crossover, and at the end of the study. Samples were frozen immediately at −80 °C. Twenty-six patients participated in the study and their characteristics are given in Table 1. Euthyroidism had been obtained for 24 months (6–100). Fourteen started on T_4_/T_3_ combination and 12 on T_4_ monotherapy, followed by a crossover after 3 months. The study was approved by the local ethics committee, and the study was registered in www.clinicaltrials.gov (2007-09-18, Study ID: T_4_-T_3_ hypothyroidism).

### Biochemical parameters

SHBG was measured by ELISA (DRG, International, Inc., Springfield, NJ, USA) (intra-assay coefficient of variation (CV): 5%), NT-proBNP was measured by a chemiluminescence enzyme immunoassay (Immulite 2500) (intra-assay CV <5%), PINP was measured by RIA (Orion Diagnostica, Espoo, Finland) (intra-assay CV 8%), and thyroid function parameters were measured by chemiluminescence enzyme immunoassays (Immulite 2500), and intra-assay CV were TSH: 5%; T_3_: 7%; T_4_: 5%; T_3_-uptake: 4%. Free T_4_ and T_3_ indices (FT_4_I and FT_3_I) were calculated by multiplying the total hormone concentration with the T_3_ uptake test.

### Statistical analyses

Non-parametrical statistical analyses, such as Friedman repeated measures ANOVA on ranks (Friedman test) and Wilcoxon test, were used. The significance level was 0.05.

## Results

**Figure 1:**
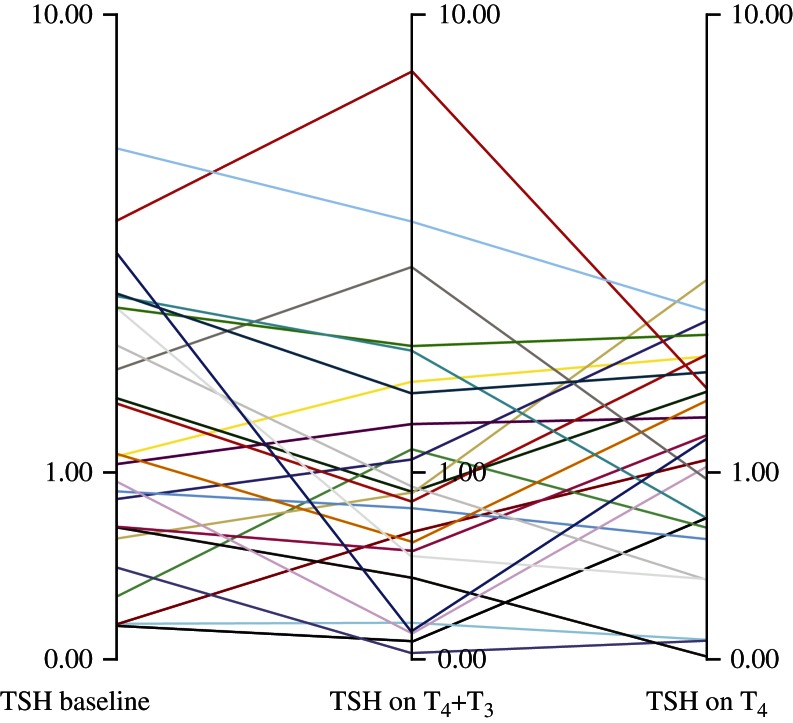
Individual serum TSH levels (mU/l) on a log-scale. Values are given in median.

TSH levels neither changed over time (baseline vs treatment: T_4_/T_3_ combination: *P*=0.101; T_4_ monotherapy: *P*=0.322) nor between groups (*P*=0.534) (Table 2). Individual TSH levels are presented in Fig. 1. The ratio FT_4_I/FT_3_I was calculated and was found to be similar before randomization (in median 80) and after T_4_ monotherapy (78), whereas the ratio decreased remarkably (29) during combination therapy reflecting both decreasing FT_4_I and increasing FT_3_I.

SHBG levels were different between groups testing for trends (*P*=0.011) due to significant higher SHBG in the T_4_/T_3_ combination group. Similarly, PINP levels also differed between groups (*P*=0.001) due to its higher level in the T_4_/T_3_ combination group. NT-proBNP levels were not affected by the different treatment regimens.

## Discussion

The availability of active thyroid hormones (mainly, T_3_) is a complex process. These hormones reach tissues via circulation, active transmembrane transport, intracellular regulatory metabolism, through three active deiodinases: types 1- and 2-deiodinase activate T_4_ into T_3_ and type 3-deiodinase inactivate T_3_, and finally, by binding to specific thyroid hormone nuclear receptors (1). Different tissues have different metabolic pathways, making it difficult to quantify the actual effect of the total thyroid hormones on a specific tissue at a given time (1).

In this study, we aimed to examine whether T_4_/T_3_ combination therapy vs conventional T_4_ monotherapy resulted in a change in the sensing of the local thyroid hormone action on different peripheral tissues. We measured SHBG, PINP, and NT-proBNP as all these markers have been shown to be sensitive parameters of changes in peripheral thyroid hormone action. SHBG levels reflected stimulation of hepatic function (11), PINP levels reflected a stimulation of collagen production during bone formation (12), and NT-proBNP levels reflected the direct stimulation of cardiomyocytes (13, 14).

Our data demonstrated that both SHBG and PINP increased when the patients received T_4_/T_3_ combination therapy compared with standard treatment with T_4_ monotherapy. However, NT-proBNP levels did not change. Thus, treatment with either of the therapies in presumably equipotent doses with respect to pituitary sensing as measured by circulating TSH levels, indeed, seems to result in different sensing of thyroid hormone action at the peripheral tissue level and in different tissues, such as hepatocytes and osteoblasts. This suggests that the two regimens have more widespread differences in intracellular thyroid hormone availability.

Understanding our findings might be related to T_3_ kinetics and T_3_ formulations used. T_3_ was given at a standard dose of 20 μg once daily in the morning. Blood samples were drawn in the morning and before the intake of the thyroid hormone, which gives ∼24-h abstinence from medicine. The short half-life time (10–15 h) of T_3_ compared with T_4_ (∼5 days) results in relative overexposure of T_3_ in the T_4_/T_3_ group vs the T_4_ only group during the early hours of absorption, and consequently, relative underexposure in the next morning when the blood sampling was performed. Although not precisely known, the plasma half-life time of especially PINP and SHBG are considerably longer than that of TSH (days vs minutes). This means that increased levels of PINP and SHBG might be due to a previous period of overexposure of T_3_ in peripheral tissues, including the absorption period. The latter probably has less effect on serum TSH as measured the next morning, which was not clarified by this study design. Our data on mental health, which was published previously, demonstrated a long-lasting beneficial effect of T_4_/T_3_ combination therapy with respect to QOL, depression, and anxiety rating scales (7). We might have unknowingly overtreated the patients for a short period during the T_3_ absorption phase, but the data on mental health point to a beneficial effect of combination therapy. Thus, the optimal replacement regimen would be to divide the dose, give a slow release preparation, or prescribe T_3_ in a dose, resulting in a fixed ratio of the actual T_4_ dose.

In a recent randomized, double-blind, crossover trial, hypothyroid subjects on stable T_4_ replacement therapy were switched to either T_4_ or T_3_ monotherapy, both given as thrice daily regimens in order to reduce excursions in T_4_ and T_3_ serum levels (15). Serum thyroid hormone levels were measured every 4 h. Even on a thrice daily regimen, serum T_3_ levels fluctuated showing individual levels above the normal range especially in the absorption periods, although mean values remained within the normal range. Thyroid hormone doses were regulated by keeping serum TSH levels similar in the two groups. The study demonstrated a distinct metabolic effect during T_3_ replacement, which was not seen during T_4_ replacement. It indicated a significant weight loss and a reduction in total and LDL cholesterol, shortened isovolumic relaxation time of the heart, and increased serum SHBG concentrations. Thus, these data also demonstrated a differential effect of T_3_ vs T_4_ on SHBG levels. At a glace, these data suggest a beneficial effect of T_3_. However, the data might also be interpreted as a result of slightly overtreatment of the subjects with T_3_ during periods of T_3_ absorption.

A previous study demonstrated that hypothyroid subjects on stable T_4_ monotherapy had lower SHBG levels than controls with similar TSH levels, and the authors hypothesized that T_3_ availability was reduced in the hypothyroid group as expressed by a high T_4_/T_3_ ratio in plasma (16). Similarly, treatment with 20 μg T_3_ once daily to euthyroid, obese subjects resulted in unaltered and normal TSH levels but an increase in serum SHBG levels (17).

Whether increased SHBG levels represent a metabolically beneficial process is not clear; however, reduced SHBG levels are associated with insulin resistance and obesity (18). In a recent study on healthy women, SHBG was suggested as a causal role of developing type 2 diabetes: the lower the levels, the higher the risk (19).

PINP levels reflect collagen production during bone formation and have been previously shown to be elevated in hyperthyroid subjects (20). Treating nontoxic goiter patients with small doses of T_4_ or T_3_ as a once daily regimen for a similar and a modest reduction in TSH levels without inducing overt hyperthyroidism resulted in increased PINP levels (12). This is in agreement with the well-known enhanced bone turnover in subclinical hyperthyroidism, both exogenous (due to T_4_ treatment) and endogenous (21). However, no difference was found in the PINP levels of patients on T_4_ vs T_3_ monotherapy (12). It is not clear whether our findings are beneficial to bone heath. Osteoblast stimulation, as evidenced by increased PINP levels, leads to increased bone formation, but also bone degradation as a secondary event, which is evident after a few months of bone stimulation (22). This is independent of menopausal status. We had measured PINP after 3 months of treatment. Therefore, bone turnover might have been stimulated at that time, which might be regarded as an adverse effect to T_3_ substitution leading to bone loss, although reversible after cessation of the T_3_ treatment (22). Thus, increased PINP levels seen after T_4_/T_3_ combination might reflect a subtle and beneficial increase in bone formation or an adverse effect on bone tissue due to increased bone turnover.

The production of NT-proBNP seems to be stimulated mainly by T_3_ in a dose-dependent manner (14). Serum NT-proBNP levels are elevated in subclinical hyperthyroidism and reduced in subclinical hypothyroidism, both normalizing when treated to obtain normal serum TSH levels (13). Thus, its secretion seems sensitive to small changes in thyroid hormone availability. Stimulation of NT-proBNP (and BNP being secreted in equipotent amounts to NT-proBNP) production is probably beneficial since BNP results in reduced cardiac after load due to vasodilatation. However, we could not find any difference between the two treatment regimens in this study.

Our main message, as discussed earlier, is that adding T_3_ to a thyroid hormone substitution regimen seems to result in a different pattern in the thyrometabolic status of different tissues compared with the traditional T_4_ monotherapy regimen. Clearly, further studies are needed in order to elucidate the potential long-term effect of T_3_ treatment either as monotherapy or in combination with T_4_.

Limitations: the number of subjects studied was relatively small, which, however, was counteracted by the study design. It was difficult to keep serum TSH stable, which is a known problem in the daily clinical work-up on T_4_-treated subjects. This meant that although mean TSH levels were kept unchanged during the two study periods, a few patients had TSH levels outside the normal range (Fig. 1). Whether this affected our results is not clear; however, we decided to include these subjects in the statistical analyses.

No washout period was inserted between the two dosing regimens as it would develop overt hypothyroidism during this period, which would probably affect the results. In our main study, presented in 2009 (7), an analysis on the QOL and depression scores between the two treatment periods did not disclose any carryover effect.

## Conclusion

Despite similar TSH levels, several peripheral tissues seem to register different T_3_ availability during T_4_/T_3_ combination substitution to hypothyroid subjects. Both hepatic function and collagen production during bone formation seem to be stimulated. Our study, as well as other recent studies, encourages the development of prolonged T_3_ formulations and testing in the clinical setting. However, the benefits of our findings are still unclear.

## Figures and Tables

**Table 1:** Clinical characteristics of the 26 l-T_4_ substituted hypothyroid subjects participating in the study (values are presented as median (range))

	**Baseline**	**T_4_ monotherapy**	**T_4_/T_3_ combination therapy**
Sex (females/males)	23/3		
Age (years)	42.5 (19–69)		
BMI (kg/m^2^)	24.8 (18–38)		
Pre/postmenopausal status	15/8		
T_4_ dose (μg/day)	132 (50–200)	125 (75–225)	75 (25–75)
T_3_ dose (μg/day)	–	–	20 (20–20)

**Table 2:** Thyroid hormone levels and peripheral markers of thyroid function in 26 l-T_4_-substituted hypothyroid subjects, treated with either T_4_/T_3_ combination of T_4_ monotherapy for 3 months in a prospective, randomized, crossover design (median (range))

	**Before randomization** (1)	**T_4_ monotherapy** (2)	**T_4_/T_3_ combination therapy** (3)	**Analysis for trend *P* value** (Friedman test)	**Paired test between groups** (Wilcoxon test)
TSH (mU/l)	1.13 (0.13–5.67)	1.18 (0.01–3.08)	0.83 (0.02–7.93)		1 vs 2: *P*=0.322
					1 vs 3: *P*=0.101
					2 vs 3: *P*=0.534
FT_4_I (units)	133 (46–168)	139 (64–200)	80 (32–191)	<0.001	1 vs 3: *P*<0.001
					2 vs 3: *P*<0.001
FT_3_I (units)	1.56 (1.03–3.28)	1.71 (1.20–4.40)	2.71 (0.95–5.20)	<0.001	1 vs 3: *P*<0.001
					2 vs 3: *P*=0.001
Ratio FT_4_I/FT_3_I	80 (45–125)	78 (33–124)	29 (9–162)	<0.001	1 vs 3: *P*<0.001
					2 vs 3: *P*<0.001
SHBG (nmol/l)	74.5 (18–254)	66.5 (19–260)	82.5 (23–260)	0.011	1 vs 3: *P*=0.015
					2 vs 3: *P*=0.041
NT-proBNP (ng/l)	63.5 (20–156)	55.5 (20–257)	56.5 (23–241)	0.446	
PINP (μg/l)	37.8 (8.1–193)	40.4 (8.5–88.9)	48.1 (13.6–171)	0.001	1 vs 3: *P*=0.08
					2 vs 3: *P*<0.001
